# Preclinical evaluation of EpCAM-binding designed ankyrin repeat proteins (DARPins) as targeting moieties for bimodal near-infrared fluorescence and photoacoustic imaging of cancer

**DOI:** 10.1007/s00259-023-06407-w

**Published:** 2023-08-29

**Authors:** Ruben D. Houvast, Nada Badr, Taryn March, Lysanne D. A. N. de Muynck, Vincent Q. Sier, Timo Schomann, Shadhvi Bhairosingh, Victor M. Baart, Judith A. H. M. Peeters, Gerard J. P. van Westen, Andreas Plückthun, Jacobus Burggraaf, Peter J. K. Kuppen, Alexander L. Vahrmeijer, Cornelis F. M. Sier

**Affiliations:** 1 Department of Surgery, Leiden University Medical Center, Leiden, the Netherlands; 2 Department of Clinical Pharmacy and Toxicology, Leiden University Medical Center, Leiden, the Netherlands; 3 Department of Radiology, Leiden University Medical Center, Leiden, the Netherlands; 4 Division of Drug Discovery and Safety, Leiden Academic Centre for Drug Research, Leiden, the Netherlands; 5 Department of Biochemistry, University of Zürich, Zurich, Switzerland; 6 Centre for Human Drug Research, Leiden, the Netherlands

**Keywords:** Molecular imaging, Fluorescence-guided surgery, Photoacoustic imaging, Colon cancer, EpCAM, DARPins

## Abstract

**Purpose:**

Fluorescence-guided surgery (FGS) can play a key role in improving radical resection rates by assisting surgeons to gain adequate visualization of malignant tissue intraoperatively. Designed ankyrin repeat proteins (DARPins) possess optimal pharmacokinetic and other properties for in vivo imaging. This study aims to evaluate the preclinical potential of epithelial cell adhesion molecule (EpCAM)-binding DARPins as targeting moieties for near-infrared fluorescence (NIRF) and photoacoustic (PA) imaging of cancer.

**Methods:**

EpCAM-binding DARPins Ac2, Ec4.1, and non-binding control DARPin Off7 were conjugated to IRDye 800CW and their binding efficacy was evaluated on EpCAM-positive HT-29 and EpCAM-negative COLO-320 human colon cancer cell lines. Thereafter, NIRF and PA imaging of all three conjugates were performed in HT-29_luc2 tumor-bearing mice. At 24 h post-injection, tumors and organs were resected and tracer biodistributions were analyzed.

**Results:**

Ac2-800CW and Ec4.1-800CW specifically bound to HT-29 cells, but not to COLO-320 cells. Next, 6 nmol and 24 h were established as the optimal in vivo dose and imaging time point for both DARPin tracers. At 24 h post-injection, mean tumor-to-background ratios of 2.60 ± 0.3 and 3.1 ± 0.3 were observed for Ac2-800CW and Ec4.1-800CW, respectively, allowing clear tumor delineation using the clinical Artemis NIRF imager. Biodistribution analyses in non-neoplastic tissue solely showed high fluorescence signal in the liver and kidney, which reflects the clearance of the DARPin tracers.

**Conclusion:**

Our encouraging results show that EpCAM-binding DARPins are a promising class of targeting moieties for pan-carcinoma targeting, providing clear tumor delineation at 24 h post-injection. The work described provides the preclinical foundation for DARPin-based bimodal NIRF/PA imaging of cancer.

## Introduction

Cancer is a leading cause of death worldwide and the incidence is increasing rapidly [1]. Despite recent therapeutic advances, curative cancer care is still based on achieving local control through radical surgical resection [2]. For most cancers, the presence of a positive resection margin (R1 resection) is associated with increased local recurrence and distant metastasis, accompanied by a reduced disease-free and overall survival [2–5]. Therefore, adequate intraoperative localization of malignant tissue is crucial for effective cancer treatment.

Intraoperatively, delineation of malignant tissue using tactile feedback is challenging and the introduction of laparoscopy and robotics has reduced this ability even further. Alternatively, surgeons can rely on intraoperative tumor imaging techniques, such as fluorescence-guided surgery (FGS) [6, 7]. FGS provides real-time tumor delineation through untargeted or tumor-targeted near-infrared fluorescent (NIRF) contrast agents which are visualized through a dedicated NIRF camera system. Clinical studies have shown that FGS indeed improves intraoperative tumor detection, regularly leading to a change of the initial surgical plan [8–10]. A limitation of using NIRF contrast is the limited NIRF light tissue penetration depth (~ 7 mm) due to photon scattering and absorption, which restricts the ability to visualize deeper-located lesions [11, 12]. Although NIRF imaging suffices for visualization of superficial lesions and resection margins, for detection of deeper lesions or resection margins, the combination with an additional real-time technique like photoacoustic (PA) imaging would be beneficial.

PA imaging via high-resolution ultrasound (US) relies on the detection of acoustic waves caused by the thermoelastic effect undergone by NIRF dyes after exposure to a nano-second pulsed NIR laser [13]. PA imaging has a higher spatial resolution than optical NIRF imaging and a deeper tissue penetration of up to 7 cm. By combining 3D information derived from PA imaging with superficial NIRF imaging, the presence of tumor lesions can be verified with increased “depth-of-view,” thereby synergistically enhancing tumor detection [14, 15].

The potential of a bimodal NIRF/PA imaging contrast agent is shaped by the careful selection of a tumor-specific biomarker in combination with a suitable targeting moiety. One promising tumor-specific target is the epithelial cell adhesion molecule (EpCAM). EpCAM is a 40 kDa-transmembrane glycoprotein expressed at the basolateral membrane of human epithelia where it plays a role in key cellular processes including cell adhesion, migration, proliferation, and differentiation [16, 17]. However, in cancer, EpCAM becomes highly and homogenously overexpressed on the entire cell membrane [17, 18]. Although originally identified in colorectal adenocarcinoma, strong overexpression of EpCAM has been described in virtually all cancer types, such as breast, lung, bladder, prostate, esophageal, gastric, and pancreatic cancer [17, 19]. With regard to EpCAM-based NIRF tumor imaging, monoclonal antibody (mAb) and mAb-derived targeting molecules have previously been evaluated by our group and others and were shown to provide high-contrast intraoperative tumor delineation of colon, breast, and prostate cancer in preclinical in vivo models [20–22].

However, the large size of mAbs limits extravasation and tissue penetration, leading to a relatively long time of 3 to 5 days between tracer injection and the optimal imaging time window [23, 24].

Consequently, the quest for a novel category of smaller, high-affinity, and easy-to-produce targeting moieties has led to the introduction of designed ankyrin repeat proteins (DARPins) [23].

DARPins (~ 14 kDa) are a novel category of synthetic consensus proteins with a randomized binding surface. They consist of four to six ankyrin repeats that are tightly folded together creating a hydrophobic core and a large, groove-like binding surface [23, 25]. Their high affinity, thermodynamic stability, solubility, low aggregation tendency, and easy engineerability have made DARPins a promising tumor-targeting alternative to mAbs [23, 26, 27]. Despite their optimal pharmacokinetics for these applications, the potential of DARPins to serve as targeting moieties for NIRF/PA imaging is still to be elucidated.

This study therefore aimed to evaluate the preclinical potential of EpCAM-binding DARPins as targeting moieties for NIRF and PA imaging of cancer. To accomplish this, the EpCAM-specific DARPins Ec4.1 and Ac2 were conjugated to NIRF dye IRDye 800CW, after which their binding and NIRF imaging potential were evaluated using in vitro and in vivo tumor models [28]. We focused on colon cancer considering the strong EpCAM overexpression in this tumor type, but consider the findings of this proof-of-concept study as extrapolatable to virtually all EpCAM-expressing cancers.

## Materials and methods

### Expression and purification of DARPins

The EpCAM-binding DARPins Ac2_M34L_cys and Ec4.1_M34L-cys (both carrying a M34L mutation and a C-terminal Gly-Gly-Cys tail) were expressed and purified by the method previously described [28–30]. Ec4.1 differs from Ec4 by a T54A mutation in a randomized position, which has decreased the dissociation rate constant by a factor 10 (N. Stefan et al., unpublished results) without changing the association rate constant. The negative control DARPin Off7 was equipped with the same C-terminal Gly-Gly-Cys tail and purified analogously [31].

### Conjugation of DARPin-800CW conjugates

DARPins Ac2_M34L_cys, Ec4.1_M34L-cys, and Off7-cys (10 mg/mL), each containing a single-cysteine residue, were treated with 10 equivalents of tris (2-carboxyethyl) phosphine (TCEP; 0.11 M in H_2_O, adjusted to pH 7 with NaOH) under an atmosphere of N_2_ for 1 h. The TCEP was removed by filtration through Zeba spin filters (Thermo Fisher Scientific, Waltham, MA, USA; MWCO 7 K) and the reduced DARPin solutions were adjusted to a concentration of 5 mg/mL with phosphate-buffered saline (PBS). Three equivalents of IRDye 800CW-maleimide (LI-COR, Lincoln, NE, Nebraska) in DMSO were added to each DARPin solution, which were left in the dark for 1–1.5 h with occasional shaking. Excess unconjugated dye was removed by double filtration through Zeba spin filters (MWCO 7 K), furnishing the mono-800CW substituted DARPins in PBS.

### Human cancer cell lines

Human colon cancer cell lines HT-29 (EpCAM-positive) and COLO-320 (EpCAM-negative) were obtained from ATCC and cultured in RPMI 1640 cell culture medium (Gibco, Invitrogen, Carlsbad, CA, USA) supplemented with L-glutamine, 25 mM HEPES, 10% fetal bovine serum (FBS; Hyclone, Thermo Fisher Scientific), and penicillin/streptomycin (both 100 IU/ml; Invitrogen). For in vivo studies, HT-29 was transfected with luciferase 2 (luc2) to allow monitoring of tumor growth using bioluminescence imaging (BLI). Absence of *Mycoplasma* was evaluated using the polymerase chain reaction. Cells were grown in a humidified incubator at 37 °C and 5% CO_2_ and subsequently detached with trypsin/EDTA (0.5% Trypsin-EDTA solution 10 × ; Santa Cruz Biotechnology, Inc, Dallas, TX, USA) when 90% confluence was reached. Viability was assessed using the trypan blue staining in 0.4% solution (Invitrogen).

### Cell-based plate assay

Colon cancer cells were grown in a 96-well plate; 20,000 cells/well in 100 µl of complete medium (Corning Costar Inc., Cambridge, MA, USA) until 90% confluency. Cells were then washed twice with PBS supplemented with 0.5% bovine serum albumin (0.5% PBSA). To evaluate DARPin binding, HT-29_luc2 and COLO-320 cells were incubated with Ac2-800CW, Ec4.1-800CW or non-binding control Off7-800CW in PBS at concentrations of 1, 10, 100, or 1000 nM for 1 h. Incubation was performed on ice and without exposure to light. Thereafter, cells were washed twice with 0.5% PBSA to wash away unbound DARPin-800CW. For competition experiments, these aforementioned steps were slightly adapted. Washed cells were preincubated with PBS, unconjugated Ac2, Ec4.1, or non-binding control Off7 at a concentration of 200 nM, followed by washing and incubation with Ac2-800CW, Ec4.1-800CW, or Off7-800CW at a concentration of 100 nM. DARPin-800CW fluorescence was measured using the Odyssey CLx Infrared Imaging System (LI-COR) using the 800 nm channel (excitation 785 nm, emission filter 812–823 nm). For cell number estimation via nuclear fluorescence, cells were permeabilized with 40–60% acetone-methanol for 5 min, washed once, and incubated with ToPro-3 iodide (1:2000, T3605, Invitrogen, California, USA) at room temperature for 10 min. After one washing step, nuclear fluorescence was quantified using the 700 nm channel of the Odyssey (excitation 685 nm, emission filter 710–730 nm). The mean fluorescence intensity (MFI) was calculated by dividing the 800-nm fluorescence signal by the nuclear 700-nm signal. Measurements were performed in triplicate.

### Flow cytometry

After detaching and viability assessment, cells were resuspended in ice-cold 0.5% PBSA at 500,000 cells/tube followed by 2 washings. Thereafter, cells were incubated with 100 nM Ac2-800CW, Ec4.1-800CW, or Off7-800CW for 1 h. After washing twice, cells were resuspended in 400 µl PBSA containing propidium iodide (1/4000) and measured on a LSRFortessa flow cytometer (BD Biosciences, Franklin Lanes, NJ, USA; 1.0 × 10^4^ living cells per tube) using FACS DIVA software version 7 (BD Biosciences). All incubation steps were performed on ice, without exposure to light. Data were analyzed using FlowJo™ (version 10.8.1, BD Biosciences).

### Chamber slides

After detachment and viability assessment, cells were transferred to an 8-well Nunc™ Lab-Tek™ II Chamber Slide (0.7 cm^2^/well, Thermo Fisher Scientific) at 40,000 cells/well. Once 90% confluence was reached, the medium was removed and the cells were washed twice in PBS for 5 min, followed by fixation with 1% paraformaldehyde at room temperature for 10 min. Next, cells were washed twice in PBS for 5 min and incubated with 200 nM Ac2-800CW, Ec4.1-800CW, or Off7-800CW on ice and without exposure to light for 1 h, followed by washing with PBS and demineralized water. Thereafter, plastic chambers were removed, and slides were dried and subsequently stained with ProLong Gold containing DAPI (Thermo Fisher Scientific). Slides were scanned using the DAPI (excitation 376–398 nm, emission filter 417–477 nm) and Cy7 channel (excitation 773–758 nm, emission filter 776–826 nm) of the Axio Scan Z1 (Carl Zeiss AG, Oberkochen, Germany). Images were analyzed using Zen Lite (version 3.5, Zeiss). Measurements were performed in triplicate.

### Animal models

Mice were kept at the Central Animal Facility of the LUMC, housing animals per EU Recommendation 2007-526-EC under specific pathogen-free conditions [19]. Six- to twelve-week-old female CD-1® Nude (Crl:*CD1-Foxn1*^*nu*^) mice (Charles River Laboratories, Wilmington, MA, USA) were subcutaneously inoculated on 4 spots on the back with HT-29_luc2 cells (5.0 × 10^5^ cells/spot; 3 mice per group). Tumor growth was monitored by a digital caliper. Orthotopic HT-29_luc2 models were induced as previously described [32]. Orthotopic tumor growth was monitored by bioluminescence imaging using the IVIS® Spectrum Preclinical In Vivo Imaging System (Spectrum, PerkinElmer, MA, USA). The local animal welfare body of the LUMC reviewed and approved all animal studies. Animals were humanely cared for in accordance with the Code of Practice Animal Experiments in Cancer Research and guidelines from Directive 2010/63/EU of the European Parliament on the protection of animals used for scientific purposes. Local standard operating procedures were followed for handling of animals.

### In vivo NIRF imaging

Once subcutaneous tumors reached approximately 50 mm^3^ in size, the mice were injected with either 3, 6, or 9 nmol of Ac2-800CW, Ec4.1-800CW, or non-binding control Off7-800CW dissolved in PBS by tail vein injection. For orthotopic tumors, tumors providing a BLI signal of  > 1.0 × 10^8^ p/sec/cm^2^/sr were regarded as suitable for imaging. Subcutaneous tumor-bearing mice were imaged at 1, 2, 4, 8, 24, 48, and or 72 h post-injection, while orthotopic tumor-bearing mice were imaged at the optimal imaging time point as determined using the subcutaneous tumor-bearing mice. All mice were imaged using both the preclinical Pearl Trilogy Small Animal Imaging System (LI-COR, 800 nm channel; excitation 785 nm, emission filter 820 nm) and the clinical Artemis NIR Imaging system (Quest Medical Imaging b.v., Middenmeer, The Netherlands; excitation 780 nm, emission filter 805 nm). Mice were kept under 2–4% isoflurane anesthesia during imaging. After the last measurement, the mice were sacrificed and the tumors and/or organs were resected, followed by imaging using the Pearl imaging system. Tumor and background MFIs were calculated by drawing a region-of-interest over the tumor area and adjacent normal tissue, respectively, and included as separate data points for analysis. Pearl images were analyzed using the Image Studio (version 5.2, LI-COR), while Quest images were analyzed using the Spectrum Capture Suite (Quest Medical Imaging b.v.) and subsequently ImageJ (version 1.50, National Institutes of Health, Bethesda, MD, USA). Tumor-to-background ratios (TBRs) were calculated using the following formula: TBR = MFI_tumor_/MFI_background_. For biodistribution analysis, organ MFIs were calculated by drawing a ROI over the resected organ.

### In vivo PA imaging

PA imaging was performed at 24 h post-injection using the Vevo 3100 Imaging System (FUJIFILM VisualSonics, Canada) as described before [33]. The system was equipped with Vevo LAZR-X cart, a Vevo LAZRTight Enclosure, and a Vevo Imaging Station. Mice were anesthetized and placed on a preheated imaging table. The MX550D transducer was used for US and PA imaging (FUJIFILM, VisualSonics; 25–55 MHz; axial resolution: 40 µm; excitation 780 nm). Images were analyzed using the Vevo LAB (version: 5.5.0, FUJIFILM, VisualSonics). Tumor-to-background ratios (TBRs) were calculated using the following formula: TBR = PA_tumor_/PA_background_.

### Histological analysis

Resected tumors were embedded in 4% paraformaldehyde and replaced by ethanol the next day, after which tumor tissues were embedded in paraffin. Four-µm-thick formalin-fixed paraffin-embedded tissue sections were deparaffinized in xylene for 15 min followed by fluorescence imaging using the Odyssey CLx Infrared Imaging System on the 800 nm channel. For immunohistochemical staining, sections were rehydrated in a series of decreasing ethanol dilutions and rinsed in demineralized water. Endogenous peroxidase was blocked with 0.3% hydrogen peroxide in demineralized water. Antigen retrieval was subsequently performed by heating sections at 95 °C for 10 min in EnVision Flex Target Retrieval Solution (pH 6.0) using PT Link (Dako, Glostrup, Denmark). After cooling in PBS, sections were incubated overnight in a humidified chamber at room temperature with 120 µL primary antibody: MOC31 (Acris antibodies, Herford, Germany; 0.06 µg/ml) and AE1/AE3 (Dako; 0.08 mg/ml) were used for, respectively, EpCAM and pan-cytokeratin. Next, slides were washed three times in PBS for 5 min and incubated with secondary goat anti-mouse EnVision antibody (Dako, K4001) at room temperature for 30 min, followed by an additional washing step. Staining was effected by incubation with 3,3-diaminobenzidine tetrahydrochloride solution (DAB, K3468, Agilent Technologies, Inc., Santa Clara, CA, USA) at room temperature for 10 min. Sections were then counterstained with Mayer’s hematoxylin solution (Sigma-Aldrich, Saint Louis, MO, USA). After dehydration in an incubator at 37 °C for 1 h, slides were mounted with Pertex (Leica Microsystems, Wetzlar, Germany). As histological reference, rehydrated slides were stained with Mayer’s hematoxylin solution (Sigma-Aldrich, Saint Louis, MO, USA) for 2 min and counterstained with eosin for 2 min, followed by dehydrating and mounting with Pertex. All slides were digitalized with the panoramic digital slide scanner and analyzed using the CaseViewer 2.4 (both 3D Histech, Budapest, Hungary).

### Statistical analyses

Statistical analyses and graph generation were performed with GraphPad Prism (version 9.3.1 GraphPad Software Inc., La Jolla, CA, USA). Differences between mean MFI and TBRs at different time points were compared using two-way ANOVA with Šídák correction for multiple comparisons. For the in vitro binding competition experiment, one-way ANOVA with Dunnett correction for multiple comparisons was used to calculate MFI differences. Differences with a *P*-value smaller than 0.05 were regarded as significant (ns: not significant. *P ≤ 0.05, **P ≤ 0.01, ***P ≤ 0.001, ****P ≤ 0.0001).

## Results

### In vitro binding of DARPin-800CW conjugates

**Fig. 1:**
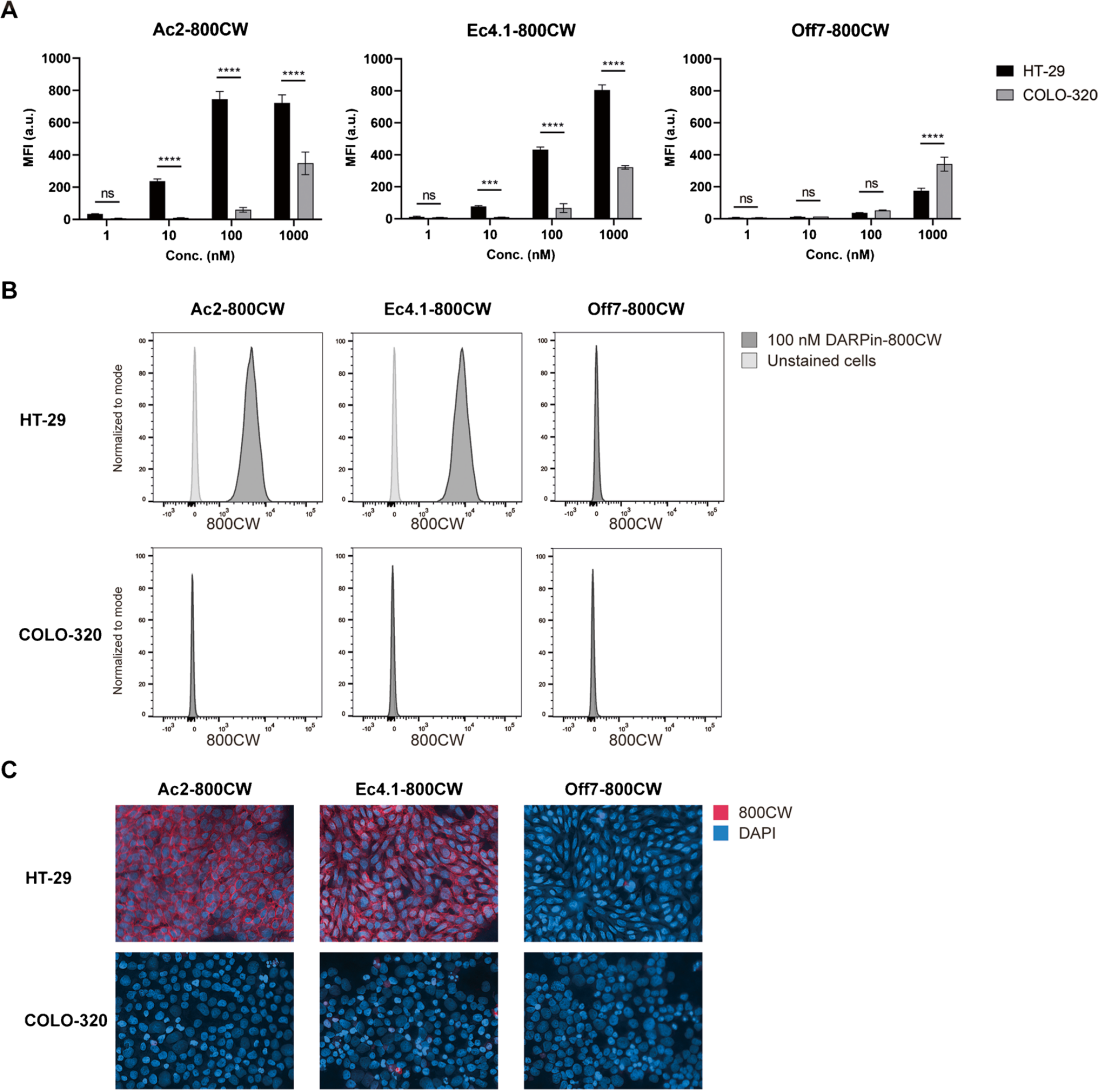
In vitro binding of EpCAM DARPin-800CW conjugates. **A** Binding of Ac2-800CW, Ec4.1-800CW, and Off7-800CW to HT-29 (black), and COLO-320 (grey) colon cancer cell lines at various concentrations using cell-based plate assays. Experiments were performed in triplicate. **B** Binding of Ac2-800CW, Ec4.1-800CW, and Off7-800CW (each at 100 nM) to HT-29 and COLO-320 cells using flow cytometry on the 800 nm channel. Dark grey curves display DARPin-800CW binding, whereas light grey curves represent unstained cells. **C** Immunofluorescence analysis of Ac2-800CW, Ec4.1-800CW, and Off7-800CW binding to HT-29 and COLO-320 cells. The 800CW signal representing DARPin-800CW localization is displayed in red. DAPI stained nuclei are displayed in blue. ns, not significant. ***P < 0.001, ****P < 0.0001

EpCAM-binding DARPins Ac2 and Ec4.1, an affinity-improved version of Ec4, and the negative control DARPin Off7 were successfully conjugated to IRDye 800CW, with the absence of free dye in the conjugate solution verified via sodium dodecyl sulfate–polyacrylamide gel electrophoresis (not shown). Next, binding to EpCAM-positive HT-29 and EpCAM-negative COLO-320 cell lines was evaluated in vitro. Using cell-based plate assays, a concentration-dependent increase in 800 nm mean-fluorescence intensity (MFI) was observed for Ac2-800CW and Ec4.1-800CW on HT-29 cells, and a significantly lower signal on EpCAM-negative COLO-320 cells (Fig. 1A). In contrast, Off7-800CW MFI did not show a substantial concentration-dependent MFI increase on either HT-29 or COLO-320 cells. Therefore, Ac2-800CW and Ec4.1-800CW specifically bind to EpCAM-positive HT-29 cells, while Off7-800CW does not. While the specific binding of Ac2 and Ec4.1 has been shown before, the present experiments show that 800CW conjugation neither sterically interferes with binding nor does it induce non-specific binding through a hydrophobic effect [28]. Binding specificity was confirmed on single cells using flow cytometry, which showed a substantial right-shift for Ac2-800CW and Ec4.1-800CW on HT-29 cells, but not on COLO-320 cells, thereby validating the observed binding specificity of Ac2-800CW and Ec4.1-800CW (Fig. 1B). As expected, Off7-800CW did not show any right-shift for either cell line.

Immunofluorescence microscopy was subsequently performed on cell-based chamber slides to evaluate the localization of DARPin-800CW binding on HT-29 and COLO-320 cells, which showed that Ac2-800CW and Ec4.1-800CW were present on the cell membrane of HT-29 cells, while neither tracer bound to COLO-320 cells (Fig. 1C). Again, Off7-800CW did not bind to HT-29 nor COLO-320 cells.

### In vitro binding competition of DARPin-800CW conjugates

**Fig. 2:**
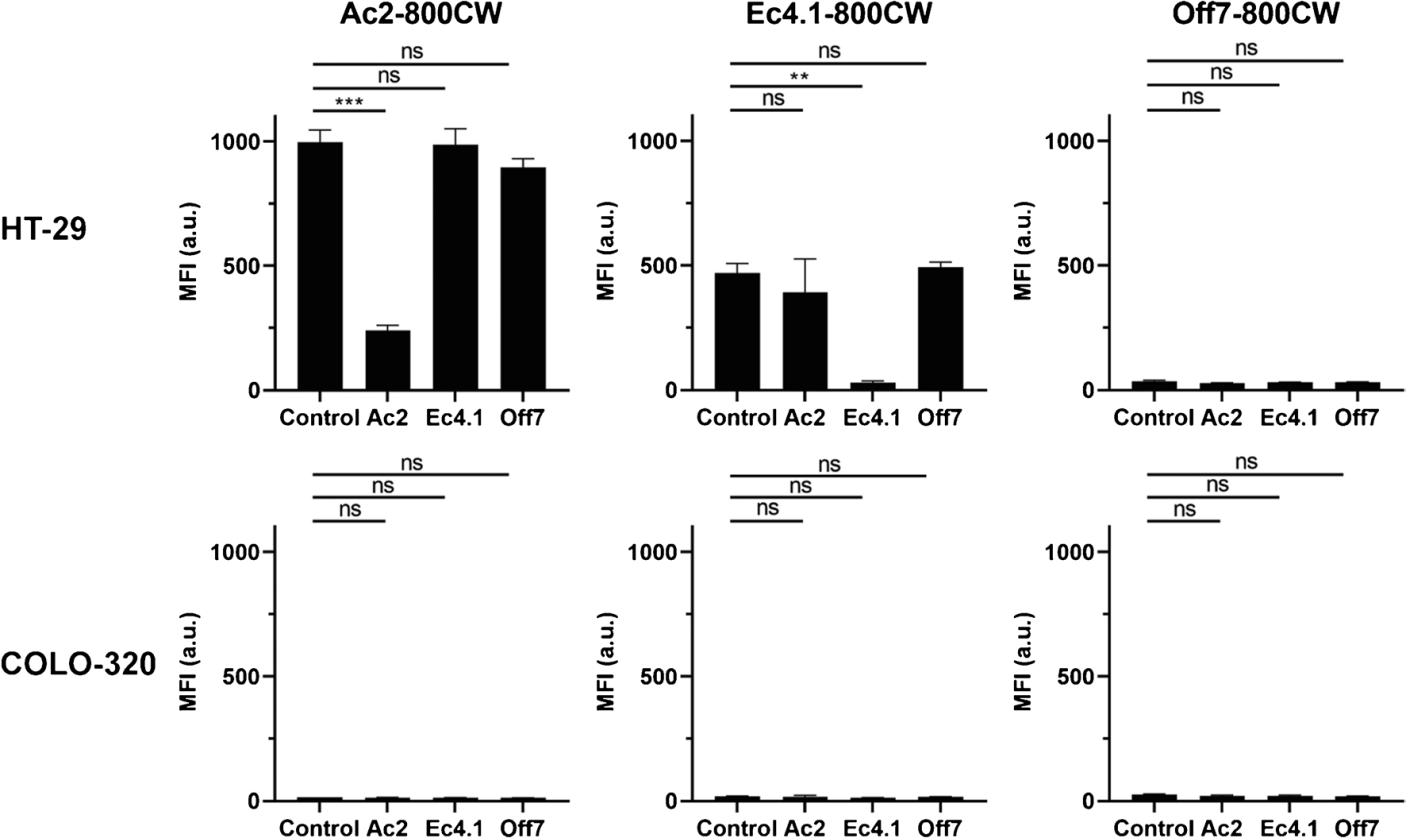
In vitro binding competition of DARPin-800CW conjugates on HT-29 and COLO-320 cells. Cells were preincubated with PBS (control), unconjugated Ac2, Ec4.1, or Off7 (each at 200 nM), followed by incubation with DARPin-800CW conjugates (100 nM). a.u, arbitrary units; MFI, mean fluorescence intensity; ns, not significant. **P < 0.01, ***P < 0.001. Experiments were performed in triplicate

To evaluate the differential epitope specificity of DARPin-800CW conjugates, in vitro binding competition between Ac2-800CW, Ec4.1-800CW, and Off7-800CW was assessed on HT-29 and COLO-320 cells using a plate assay. While Ac2-800CW and Ec4.1-800CW showed competition with their unconjugated counterpart on HT-29 cells, competition between Ec4.1 and Ac2 was absent, confirming that the two DARPins target different EpCAM epitopes [28], also when conjugated to 800CW (Fig. 2). Competition of both EpCAM-targeting DARPins by Off7 was not significant. Moreover, no binding and/or competition was found for all DARPin-800CW conjugates on COLO-320 cells. Based on the above, HT-29 was selected as a suitable EpCAM-positive cell line for in vivo experiments.

### In vivo dose and time window optimization of DARPin-800CW conjugates

**Fig. 3:**
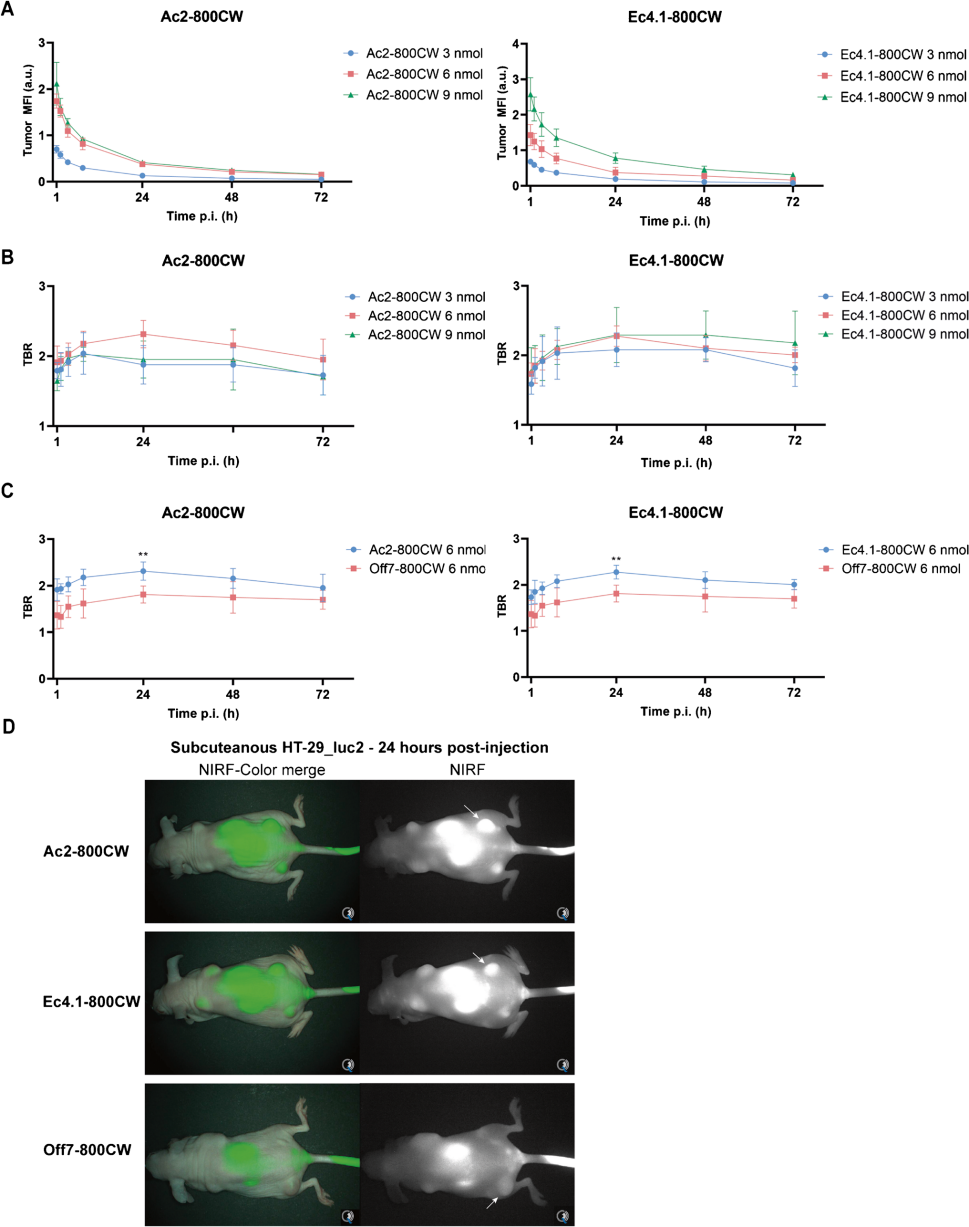
In vivo dose and time window optimization of DARPin-800CW conjugates. **A** Tumor MFIs and TBRs as a function of time after intravenous administration of 3, 6, or 9 nmol Ac2-800CW or Ec4.1-800CW in subcutaneous HT-29_luc2 tumor-bearing mice. **B** TBRs as a function of time after intravenous administration of 6 nmol Ac2-800CW, Ec4.1-800CW, and negative control tracer Off7-800CW in subcutaneous HT-29_luc2 tumor-bearing mice. **C** NIRF-color merge and NIRF images of subcutaneous HT-29_luc2 tumor-bearing mice at 24 h post-injection of Ac2-800CW, Ec4.1-800CW, or Off7-800CW. Images were captured using the clinical Artemis NIRF imager at a similar exposure time of 150 ms, allowing real-time imaging. White arrows indicate an example of a representative tumor. NIRF, near-infrared fluorescence; p.i., post-injection; TBR, tumor-to-background ratio. **P < 0.01

To establish the suitable in vivo dose and time window of DARPin-800CW conjugates, HT-29_luc2 tumor-bearing mice were injected with either 3, 6, or 9 nmol of Ac2-800CW or Ec4.1-800CW using tail vein injection followed by NIRF imaging at 1, 2, 4, 8, 24, 48, and 72 h post-injection using the preclinical Pearl imager. The tumor MFI_max_ as measured by the Pearl imager was observed at 1 h post-injection, followed by an exponential decrease (Fig. 3A). For Ac2-800CW, no substantial tumor MFI difference was observed for the 6 and 9 nmol group, whereas for Ec4.1-800CW, the highest tumor MFI was observed with the 9 nmol dose. Next, tumor-to-background ratios (TBRs) were calculated to quantify the relative tumor MFI compared to the surrounding healthy tissue. For Ac2-800CW, the highest TBRs were observed in the 6 nmol group, while for Ec4.1-800CW, comparable TBRs were observed in the 3 nmol and 6 nmol groups (Fig. 3B). The TBR_max_, as measured by the preclinical Pearl imager, was observed in the 6 nmol group at 24 h post-injection for Ac2-800CW and Ec4.1-800CW, with 2.3 ± 0.2 and 2.3 ± 0.1, respectively. Therefore, 6 nmol and 24 h were selected as the optimal dose and imaging time point for both DARPin tracers.

### In vivo binding specificity of DARPin-800CW conjugates

To verify in vivo binding specificity, HT-29_luc2 tumor-bearing mice were administered with 6 nmol Ac2-800CW, Ac2-800CW, or control Off7-800CW and imaged using the preclinical Pearl and clinical Artemis NIRF imagers at 1, 2, 4, 8, 24, 48, and 72 h post-injection. At 24 h post-injection, a significantly higher TBR was found for Ac2-800CW and Ec4.1-800CW compared to Off7-800CW, suggesting in vivo specificity of both EpCAM-targeting DARPin tracers (Ac2-800CW vs. Off7-800CW: 2.3 ± 0.2 vs. 1.8 ± 0.2, *P* = 0.003; Ec4.1-800CW vs. Off7-800CW: 2.3 ± 0.1 vs. 1.8 ± 0.2, *P* = 0.003) (Fig. 3C). As shown in Fig. 3D, HT-29_luc2 tumors can be clearly delineated using the clinical Artemis NIF imager after injection of Ac2-800CW and Ec4.1-800CW, while tumors can be less clearly localized using Off7-800CW. Moreover, kidney uptake was pronounced for all EpCAM-targeting DARPin tracers.

### In vivo NIRF imaging potential of DARPin-800CW conjugates

**Fig. 4:**
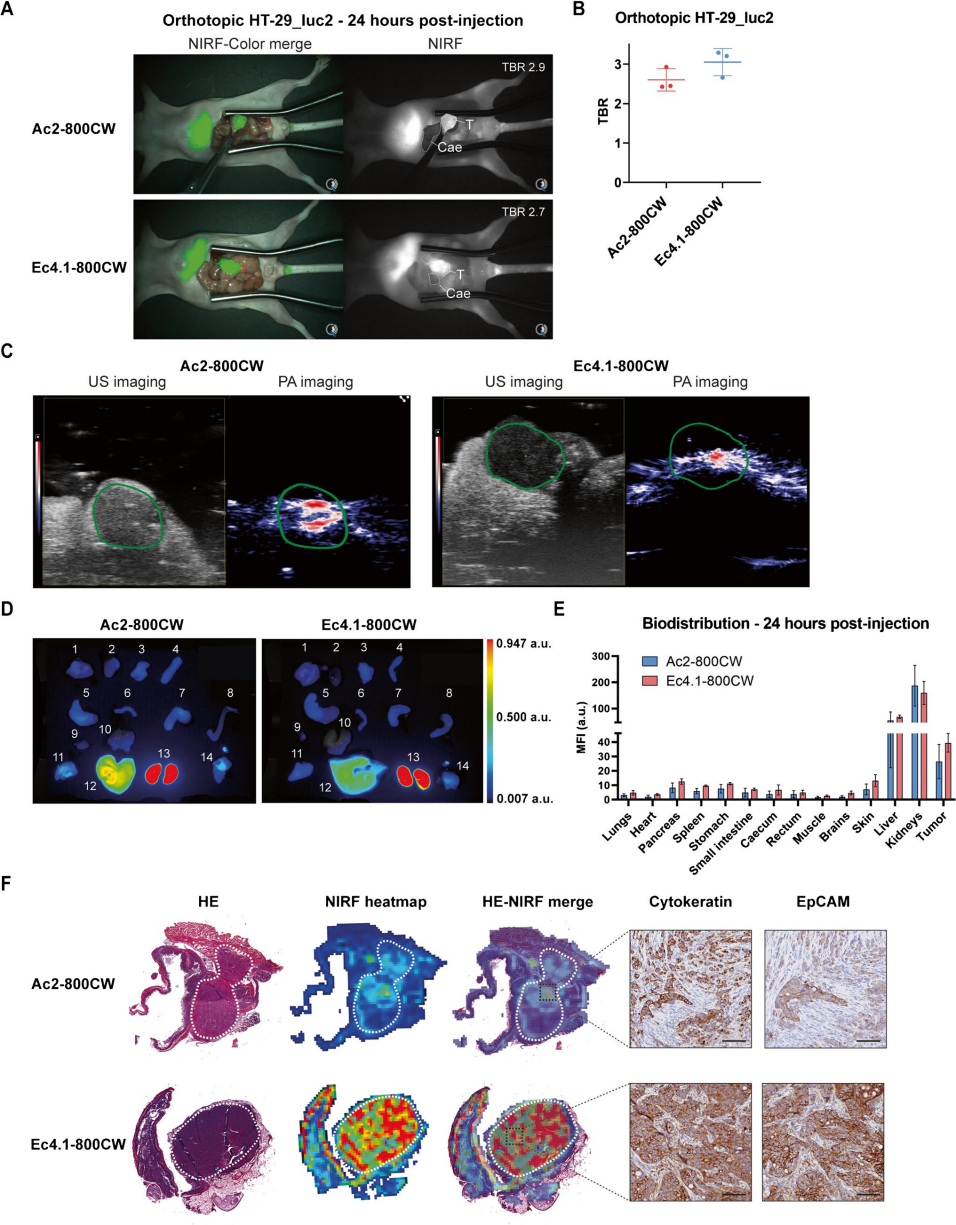
In vivo NIRF imaging and PA imaging using DARPin-800CW conjugates. **A** NIRF-color merge and NIRF images of orthotopic HT-29_luc2 tumor-bearing mice at 24 h post-injection of Ac2-800CW or Ec4.1-800CW. Images were captured using the clinical Artemis NIRF imager at an exposure time of 150 ms. “T” indicates the tumor localization, while “Cae’” indicates the corresponding background tissue (caecum). Mouse-specific TBRs are indicated in white in the right-upper quadrant of the NIRF images. **B** TBRs of orthotopic HT-29_luc2 tumors 24 h after intravenous administration of 6 nmol Ac2-800CW or Ec4.1-800CW as measured using the clinical Artemis NIRF imager. Mean TBRs are represented by the horizontal line together with their error bars representing the standard deviation. **C** Representative US and PA images of orthotopic HT-29_luc2 tumor-bearing mice at 24 h post-injection of Ac2-800CW or Ec4.1-800CW. Images were captured using a penetration depth of approximately 1.5 cm. Tumors are delineated with a green line. **D** Biodistribution in orthotopic HT-29_luc2 tumors and healthy organs of mice at 24 h post-injection of Ac2-800CW or Ec4.1-800CW. 1: lungs, 2: heart, 3: pancreas, 4: spleen, 5: stomach, 6: small intestine, 7: caecum, 8: rectum, 9: muscle, 10: brain, 11: skin, 12: liver, 13: kidneys, and 14: tumor. **E** Macroscopic fluorescence biodistribution of orthotopic HT-29_luc2 tumors and healthy organs at 24 h post-injection of Ac2-800CW or Ec4.1-800CW (Pearl imager). **F** HE staining, 800 nm heatmap and merge, as well as cytokeratin and EpCAM stainings of sequential tissue sections derived from orthotopic HT-29_luc2 tumors at 24 h post-injection of Ac2-800CW or Ec4.1-800CW. Tumors are delineated by dashed white lines. HE-NIRF and cytokeratin-EpCAM images are taken at × 2 and × 15 magnification, respectively. Scale bars represent 100 µm. a.u, arbitrary units; HE, hematoxylin-eosin; MFI, mean fluorescence intensity; NIRF, near-infrared fluorescence; PA, photoacoustic; TBR, tumor-to-background ratio; US, ultrasound

To evaluate the in vivo NIRF imaging potential of Ac2-800CW and Ec4.1-800CW in a more clinically relevant colon cancer model, mice were orthotopically inoculated with HT-29_luc2 tumors in the caecum and injected with 6 nmol Ac2-800CW or Ec4.1-800CW. For both tracers, orthotopic HT-29_luc2 tumors could be localized with high contrast at 24 h post-injection using the clinical Artemis NIRF imager (Fig. 4A). Mean Pearl TBRs of 4.2 ± 0.7 and 5.3 ± 0.5 were observed for Ac2-800CW and Ec4.1-800CW, respectively. Using the clinical Artemis NIRF imager, slightly lower mean TBRs of 2.6 ± 0.3 and 3.1 ± 0.3 were observed for Ac2-800CW and Ec4.1-800CW, respectively (Fig. 4B).

### In vivo PA imaging potential of DARPin-800CW conjugates

To establish the potential of DARPin-800CW conjugates as tracers for bimodal NIRF/PA imaging, PA imaging using Ac2-800CW or Ec4.1-800CW was performed in orthotopic HT-29_luc2 tumor-bearing mice at 24 h post-injection. As shown in Fig. 4C, PA signal is located inside the tumor lesions with high intensity for both Ac2-800CW and Ec4.1-800CW, while PA signal in surrounding tissues is limited. PA imaging TBRs of 2.7 and 2.3 were observed for Ac2-800CW and Ec4.1-800CW, respectively.

### Biodistribution and histological analysis

To verify the biodistribution of the tracers*,* tumors and organs were resected at 24 h post-injection followed by NIRF imaging. For both Ac2-800CW and Ec4.1-800CW, biodistribution analysis showed higher fluorescence signal in excretory organs such as the kidneys and liver than in tumor tissues. The tumor MFI for Ac2-800CW (26 ± 12) was lower compared than that of Ec4.1-800CW (39 ± 6), although this difference was not statistically significant (95% CI: − 9, 35; *P* = 0.17). Macroscopic fluorescence allowed clear tumor visualization for both tracers with low fluorescence signal in remaining healthy organs (Fig. 4D, E).

Histological analysis showed that NIRF signals for both Ac2-800CW and Ec4.1-800CW largely overlapped with microscopically identified tumor areas, as well as cytokeratin and EpCAM staining, thereby confirming binding specificity of both tracers and indicating complete tumor penetration (Fig. 4F). As outlined above, intratumoral fluorescence of Ac2-800CW was lower than Ec4.1-800CW fluorescence.

## Discussion

Fluorescence-guided surgery can play a key role in improving radical resection rates by assisting surgeons with intraoperative visualization of malignant tissue. The quest for adequate tumor-targeting moieties for FGS tracers has shifted from antibodies towards strategically designed targeting molecules with optimal pharmacokinetics for in vivo imaging, such as DARPins. Using real-time NIRF imaging and PA imaging, we showed that EpCAM-binding DARPins Ac2-800CW and Ec4.1-800CW provided high-contrast tumor delineation in a clinically relevant in vivo model at 24 h post-injection, accompanied by low signals in healthy surrounding organs. This study thereby provides the first preclinical substantiation that EpCAM-binding DARPins are promising targeting molecules for NIRF and PA imaging of cancer. Considering the strong abundance of EpCAM in a wide variety of epithelial cancer types, EpCAM-targeted DARPin-based NIRF/PA imaging tracers may be deployed in a broad, pan-carcinoma clinical context.

Intraoperatively, combining NIRF with PA imaging provides a powerful diagnostic and screening tool, allowing detection of malignant tissue located beyond NIRF imaging’s penetration capability using a single-contrast agent injection. Once a lesion is identified and approached guided by PA imaging, NIRF imaging allows tumor identification and removal with higher accuracy by overlaying the actual surgeon’s view with real-time fluorescence. The synergy between PA and NIRF imaging thus provides an improved intraoperative tumor imaging approach, where the strengths of each modality complement and compensate for their individual limitations. Several studies have successfully described the use of 800CW-based contrast agents for bimodal NIRF/PA imaging [33, 34]. Intraoperatively, Tummers et al. demonstrated a 3.7-fold higher mean PA signal in primary pancreatic cancer lesions compared to normal pancreatic tissue using the anti-EGFR tracer cetuximab-800CW, providing the first clinical evidence of the combined NIRF/PA imaging approach. Despite these promising findings, routine implementation is hampered by, among others, the clinical availability of PA imaging systems [35]. In contrast to NIRF/PA imaging, research into DARPins as tumor imaging agents has primarily focused on nuclear imaging, which has already yielded multiple encouraging results [28, 36–40]. Recently, a first-in-human study evaluating the anti-HER2 DARPin tracer ^99m^Tc-(HE)_3_-G3 for SPECT imaging of breast cancer reported a favorable safety and tolerability profile, and it showed clear visualization of both primary and metastatic HER2-positive lesions (NCT04277338) [41]. Interestingly, clinically defined HER2-negative tumors could also be visualized, albeit with lower contrast. Vorobyeva et al. evaluated, in a preclinical setting, the PET imaging potential of the EpCAM-binding DARPin Ec1 conjugated to [^125^I]I-PIB in a human ovarian cancer xenograft model and observed a tumor-to-blood ratio of 19 at 6 h post-injection, which increased to 31 at 24 h post-injection, thereby providing high-contrast tumor localization [38]. Although lower TBRs were achieved using NIRF instead of radiation, we observed a TBR increase to  > 2 in the subcutaneous model until 24 h post-injection, providing clear tumor localization. Obviously, tumor-to-blood ratios from nuclear imaging studies cannot be directly compared to tumor-to-background tissue ratios in NIRF imaging. This is largely caused by the presence of endogenous autofluorescence and NIR light absorption/scattering which can increase background signal, decreasing the TBR [42–44]. Of note, TBRs in the range from 2 to 3 are typically observed using NIRF-labeled, tumor-targeted nanobodies, which share similar pharmacokinetic properties with DARPins, further substantiating our findings [45, 46]. Moreover, even though Off7-800CW binding was not observed in vitro, some tumor fluorescence was observed in vivo, albeit at lower levels compared to Ec4.1/Ac2-800CW. The phenomenon that untargeted tracers show low, non-specific intratumoral uptake in human tumors grown in mice is common and attributed to the enhanced permeability and retention (EPR) effect [47].

Besides the contrast between the primary tumor and direct background (TBR), sufficient contrast between other healthy organs and common (distant) metastatic sites is crucial to decrease false-positivity and allow adequate NIRF/PA imaging-based intraoperative staging [48, 49]. For colon carcinoma, common metastatic sites are the liver and peritoneum [50]. Our biodistribution analysis at 24 h post-injection showed high fluorescence in the liver and kidneys, in line with previous reports on DARPin-based imaging [38, 51]. As high liver fluorescence was observed for both DARPin tracers, visualization of hepatic metastases could potentially be impaired in the clinical setting, which should be considered when choosing suitable applications. In contrast, the high kidney fluorescence, which can be attributed to renal clearance of the construct, will be reduced in humans due to the presence of a more pronounced retroperitoneal perinephric fat layer along with Gerota’s fascia [45]. Moreover, renal metastases are rarely observed for any cancer type [52]. Nonetheless and in line with previous literature, DARPin-800CW conjugate fluorescence in peritoneal organs has been found to be low, theoretically allowing visualization of EpCAM-expressing peritoneal depositions using both DARPins tracers [37, 38, 40].

The use of mAbs has dominated the molecular imaging field for years as the first targeting molecule-of-choice [53, 54]. Despite their favorable stability, specificity, and target affinity, mAb-based tumor imaging is complicated by high costs, limited extravasation, and poor tissue penetration, resulting in a relatively long time frame (3 to 5 days) between tracer injection and imaging [23, 24]. The use of smaller targeting molecules may improve extravasation and tissue penetration and shorten the time between injection and imaging; however, their size reduction should be compensated by enhanced target affinity (K_D_), in order to achieve similar tumor uptake compared to larger molecules [55, 56]. Despite relevant affinity differences between Ac2 (K_D_: 130 nM) and Ec4.1 (K_D_: 0.2 nM, ca. tenfold improved over the previously published Ec4, cf. “Materials and Methods” [28]), we found that TBRs and tumor MFIs of both DARPin tracers were sufficiently high to allow adequate tumor visualization. Nonetheless, both TBRs and MFIs were somewhat higher for the high-affinity Ec4.1, albeit only at the border of statistical significance. Of note, Ac2 and Ec4.1 affinities are comparable or higher than those of therapeutic EpCAM mAbs, such as adecatumumab (K_D_: 91 nM) or edrecolomab (K_D_: 1530 nM) [57].

In line with these findings, Zahnd et al. [58] systematically investigated the influence of molecular mass and affinity on tumor accumulation of DARPins. A strong correlation of tumor accumulation with affinity was found for these small proteins, when accumulation was evaluated by radioactivity accumulation as a function of time. Interestingly, increasing the size of the DARPins to 30 kDa resulted in significantly lower tumor accumulation after 24 h, similar to the lower values observed for scFvs, whereas valency as such had no influence on accumulation for molecules with already very high affinity [58]. For larger proteins (such as PEGylated DARPins) affinity became less important. In modelling studies, these experimental findings were completely replicated and explained by the need to avidly retain molecules of fast diffusion [59].

Although the potential effect of IRDye800CW conjugation on affinity was not quantified, the retained specificity is consistent, with the dye not interacting with the target nor impeding the interaction. Previous studies have already indicated that DARPin selectivity and affinity were retained after conjugation [30, 58, 60]. The quantitative influence of affinity and size on total accumulation, however, strongly depends on the tumor model used, regarding accessibility (orthotopic versus subcutaneous) and target expression level.

The fact that DARPins can easily be equipped with a free and unique C-terminal cysteine moiety, to enable site-specific labeling, is an important advantage of recombinant proteins above conventional, non-recombinant antibodies. Traditionally, mAbs are conjugated in a random manner using N-hydroxysuccinimide ester chemistry to link the dye to primary amino groups, generating a heterogenous conjugate in which individual mAbs contain a varying number of dye molecules and exhibit different pharmacokinetics [61–63]. Site-specific labeling, as used for DARPins, generates homogenous conjugates and prevents steric hindrance of the antigen-binding domain as well as quenching of fluorescence due to high localized fluorophore density [63, 64]. As both Ac2-800CW and Ec4.1-800CW allowed clear visualization of malignant tissue using a clinical NIRF camera system, no detrimental effect of site-specific conjugation was observed.

This study has some limitations. First, any in vivo tumor model is only an approximation of clinical practice. While EpCAM is expressed in most normal human epithelia, mice do not naturally express the human EpCAM protein, which might lead to an overestimation of the TBR [65]. However, previous research has shown that EpCAM is overexpressed up to 1000-fold on human tumor tissue compared to healthy tissue, thereby compensating for this potential overestimation [19, 21, 66]. Furthermore, the level of heterogeneity in human carcinomas is not replicated well in our in vivo model and therefore the extent of tumor penetration and diffusion of the tracers cannot be extrapolated. Since the amount of extracellular matrix is inversely correlated with the tumor penetration potential of targeting molecules, the tumor penetration capacity by DARPins reported herein could be reduced in clinical practice [55]. Even the use of clinically relevant tumour models, such as patient-derived xenografts or complex co-culture models could not compensate for this issue [67, 68]. Secondly, it is possible that the optimal time window was outside the measured imaging times. However, ethical standards for animal care limited the number of possible measurements. Therefore, imaging times were chosen based on their clinical practicality. Of note, a time frame of 24 h between injection and imaging has been extensively used in clinical practice for NIRF imaging of liver metastasis using ICG and was found to be practical [69].

Our experiments confirmed that Ac2 and Ec4.1 target different EpCAM epitopes with different affinity. Because high affinity is not per se the most important characteristic for tumor targeting, future research could therefore focus on the development of a bivalent Ac2-Ec4.1 DARPin dimer or other construct that may have even better binding potential for tumor-associated EpCAM. However, the opposing effects of avidity and hindered diffusion with the larger size [58] will require an experimental testing of this strategy. The flexible engineerability of DARPins allows for the creation of additional conjugation sites, enabling simultaneous conjugation with additional NIRF dyes or (radio)labels. This may provide opportunities for dual-labeled DARPins that may be used for trimodal NIRF/PA/nuclear imaging and/or therapeutic applications via one single administration. For instance, Van Den Brand et al. evaluated the potential for photodynamic therapy of IRDye700DX-conjugated EpCAM-binding DARPins Ac2 and Ec1 and showed effective in vitro cytotoxicity on EpCAM-positive human ovarian cancer cell lines [70]. Lastly, considering the clinical availability of IRDye 800CW, a rapid clinical translation of both EpCAM-binding DARPin tracers evaluated herein is feasible.

## Conclusion

To conclude, our findings show that bimodal NIRF/PA imaging using EpCAM-binding DARPin tracers Ac2-800CW and Ec4.1-800CW allows for clear colon tumor delineation at a rapid and clinically practical time window of 24 h post-injection. Thanks to both the tumor-specific expression pattern of EpCAM and the optimal pharmacokinetics and flexible manufacturability of DARPins, EpCAM-binding DARPins form a promising class of pan-carcinoma targeting agents. This study provides the preclinical foundation for DARPin-based bimodal NIRF/PA imaging of cancer and paves the way for further optimization, evaluation, and clinical translation of such agents.

## Data Availability

The datasets generated during and/or analyzed during the current study are available from the corresponding author on reasonable request.
